# Supplementary figures and images for: Therapeutic Effects of IL-1RA against Acute Bacterial Infections, including Antibiotic-Resistant Strains
Source: Pathogens. 2023 Dec 31;13(1):42. doi: 10.3390/pathogens13010042 (PMC10820880; doi:10.3390/pathogens13010042)

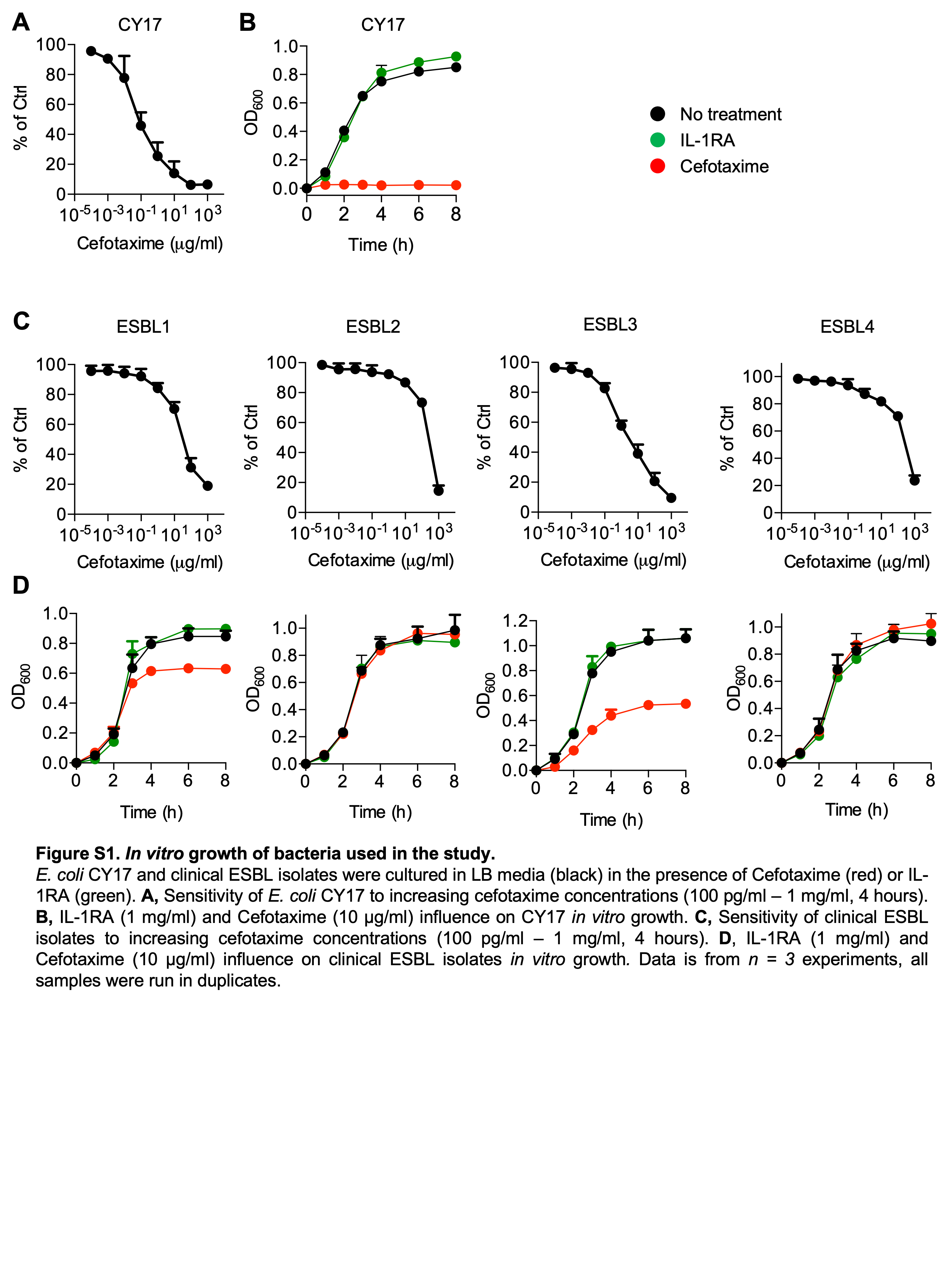

Supplement: Supplementary file 1 [file pathogens-13-00042-s001.zip › Figure S1.png]

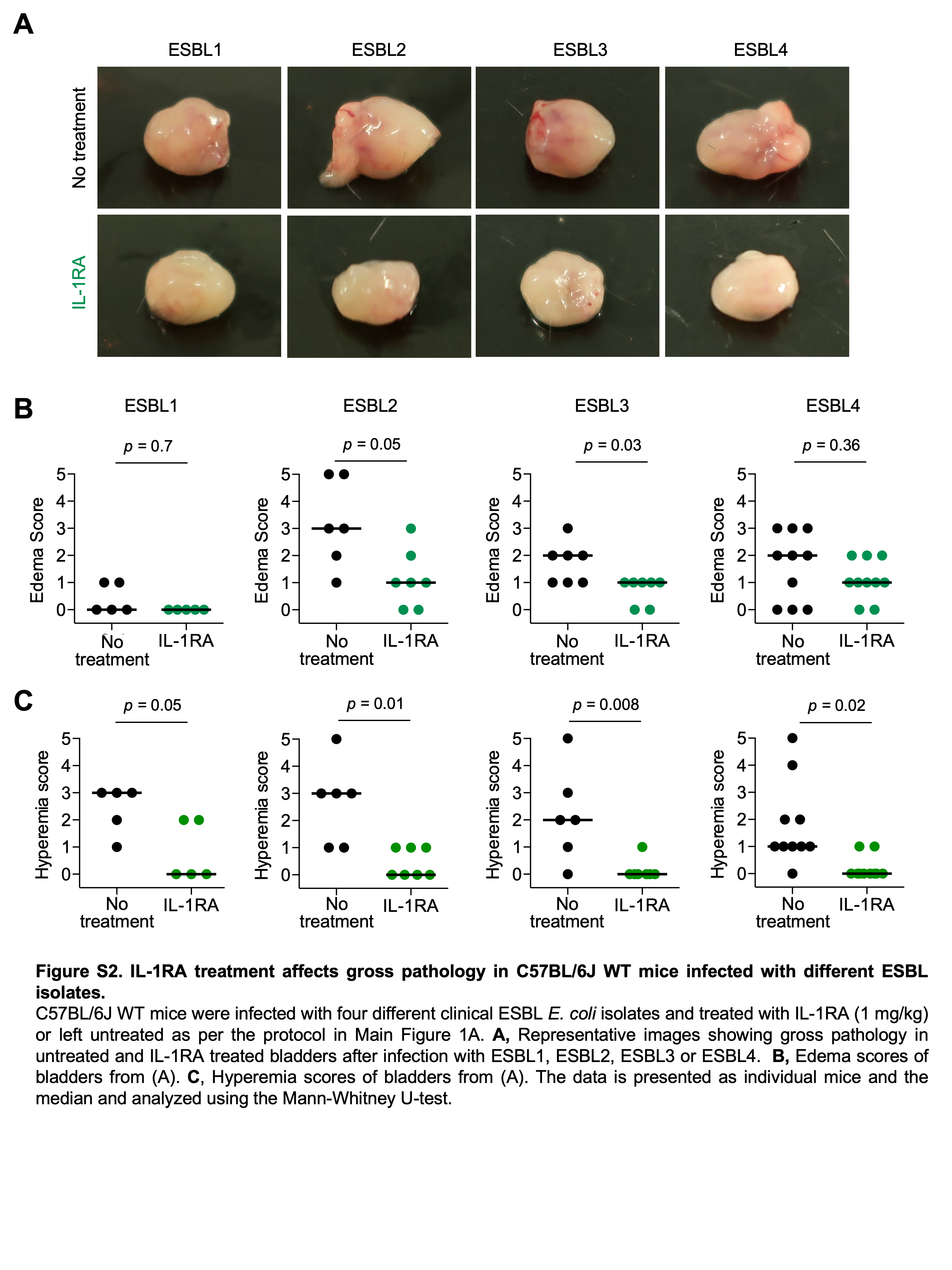

Supplement: Supplementary file 1 [file pathogens-13-00042-s001.zip › Figure S2.png]

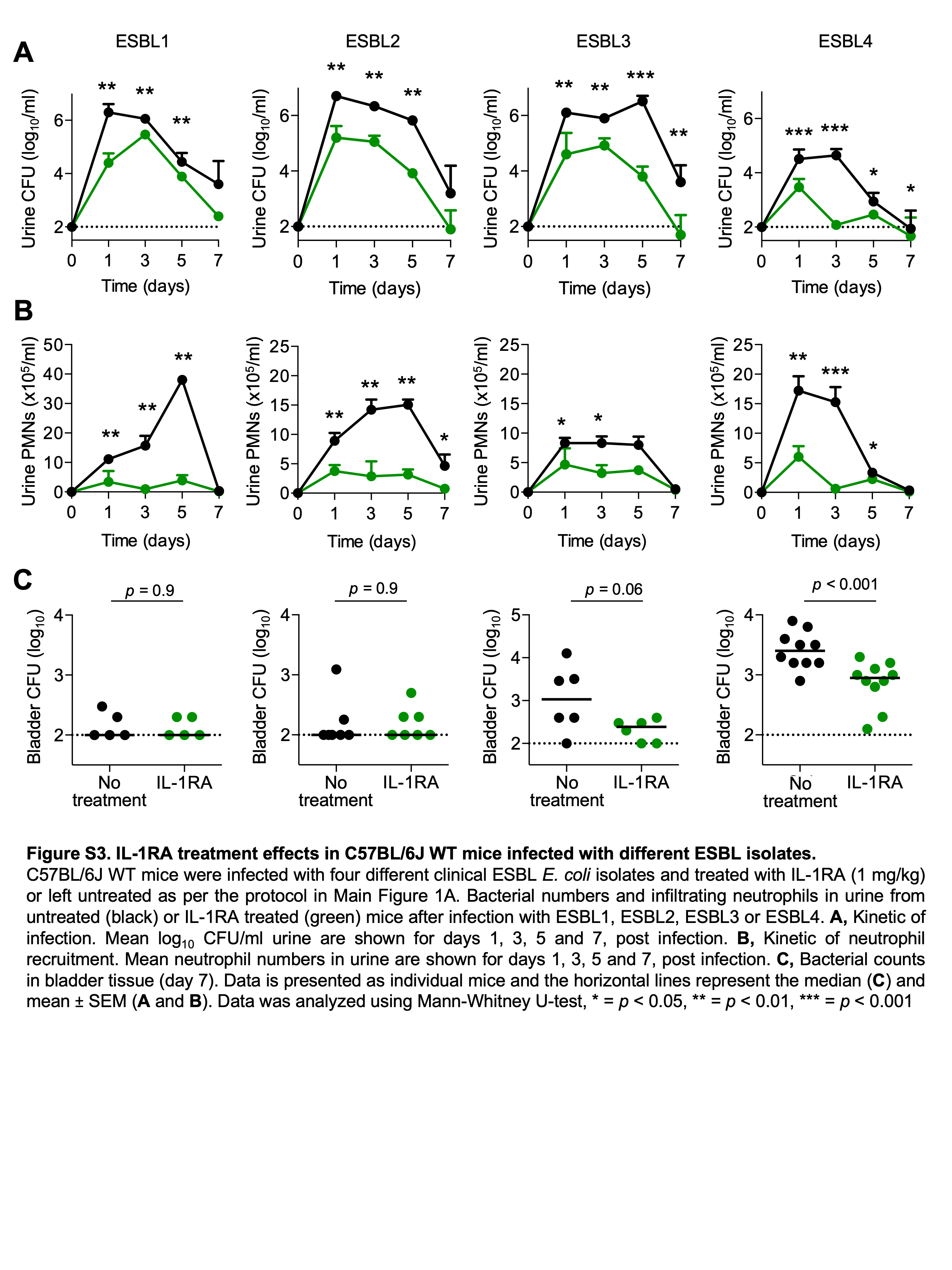

Supplement: Supplementary file 1 [file pathogens-13-00042-s001.zip › Figure S3.png]

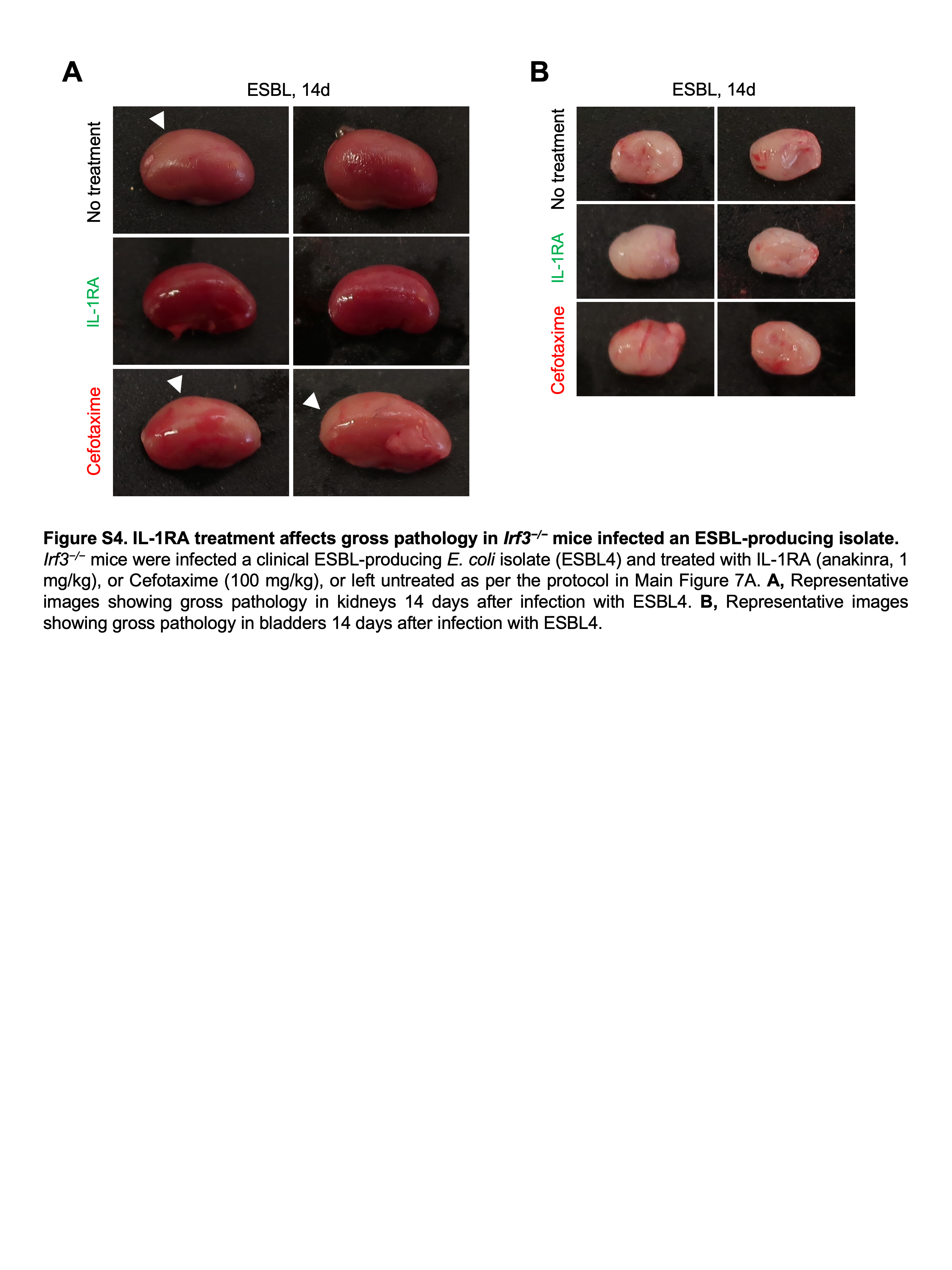

Supplement: Supplementary file 1 [file pathogens-13-00042-s001.zip › Figure S4.png]
